# Baseline determinants of adherence for drug-sensitive TB treatment in a South African prospective cohort: a focus on HIV infection and anti-retroviral therapy, clinical care access, and TB stigma

**DOI:** 10.1186/s12879-025-12304-4

**Published:** 2026-01-07

**Authors:** Adrian Steulet, Piotr Hippner, Noriah Maraba, Lauren Jennings, Israel Rabothata, Rachel Mukora, Nokhanyo Xaba, Lihle Mchunu, Kavindhran Velen, Catherine Orrell, Salome Charalambous, Katherine Fielding

**Affiliations:** 1 London School of Hygiene and Tropical Medicine, London, UK; 2 The Aurum Institute, Aurum House, Isando, Johannesburg, South Africa; 3 Desmond Tutu Health Foundation, Cape Town, South Africa; 4 Interactive Research and Development South Africa, Durban, South Africa; 5 Department of Medicine, Institute of Infectious Disease and Molecular Medicine, University of Cape Town, Cape Town, South Africa; 6 School of Public Health, University of Witwatersrand, Johannesburg, South Africa

**Keywords:** Tuberculosis, Adherence, Medication monitor, HIV, South Africa

## Abstract

**Background:**

Inadequate adherence to tuberculosis (TB) treatment increases the risk of treatment failure and recurrence. Identifying factors contributing to poor adherence could refine targeting strategies and optimize resource distribution. We examined specific individual-level factors for TB treatment adherence among adults with drug-sensitive TB, including HIV status, antiretroviral therapy, time to access clinical care, and perceived stigma.

**Methods:**

Data are from the “TB Mate” cluster-randomized trial, which evaluated a TB treatment adherence intervention across 18 public health clinics in South Africa (PACTR201902681157721). Treatment adherence was measured using smart pillboxes, with pillbox opening recorded as a proxy for the dose taken. Adults in the control group, utilizing the pillbox in silent mode, were included in this analysis. We used logistic regression to model poor adherence (< 80% pillbox engagement), and negative binomial regression to model adherence as a count of pillbox engagement. Directed acyclic graphs informed confounder selection in the models.

**Results:**

Among 1,213 participants (nine clinics) in the control group, 61.2% (742) were male, the median age was 36 years, 63% (769) were living with HIV, with 66% (507/769) on antiretroviral therapy. The median time to access clinical care was 127 min and 95% (1151/1213) reported no perceived stigmatization upon starting TB treatment. Overall 51% (614) exhibited adherence below 80%, with a geometric mean pillbox engagement of 59.6%. Living with HIV was associated with poor adherence to TB treatment, with an adjusted odds ratio of 1.68 (95% confidence interval [CI] 1.27–2.22) for < 80% adherence and an adjusted rate ratio of 0.90 (0.83–0.97) for pillbox engagement, compared to being HIV-negative. Antiretroviral therapy, time to clinical care access, and perceived stigma showed no association with either measure of adherence.

**Conclusions:**

The low adherence underscores the necessity for TB treatment support interventions, particularly among those living with HIV.

**Supplementary Information:**

The online version contains supplementary material available at 10.1186/s12879-025-12304-4.

## Introduction

Before the COVID-19 pandemic, tuberculosis (TB) was the main infectious cause of death at a global scale. South Africa is among the countries with the highest burden of tuberculosis, with an incidence of more than 500/100,000 per year [1]. In 2022, the TB treatment success rate was 77% in South Africa, while it was 85% on average worldwide [1]. Globally, 6.7% of incident TB cases in 2021 were among people living with HIV, with the proportion exceeding 50% in certain regions of Sub-Saharan Africa [1].

TB is a treatable disease with the standard drug regimen for drug-sensitive (DS) TB curing the majority of patients. However, adherence is often suboptimal for multiple reasons including the length and posology of TB treatment, side effects of TB drugs, and patient travel costs [2]. For TB treatment, sub-optimal adherence, particularly in the 2-month intensive phas, is one of the main reasons for treatment failure and development of drug resistance [2]. Various strategies have been used to improve adherence, such as reducing pill burden through combined pills and developing shorter treatment regimens [3]. Endorsed since 1993 by the World Health Organization (WHO), Directly Observed Therapy (DOT) initially increased treatment adherence. However, many trials have since failed to demonstrate improved treatment outcomes compared with unobserved treatment, and DOT is also time-consuming and costly to both patients and health care providers [4].

Routine measurement of TB treatment adherence has relied on self-reporting at, often, monthly visits to the clinic. Measuring treatment adherence is complex; recently, digital adherence technologies (DATs) such as smart pillboxes or video-supported therapy have been suggested [5]. The smart pillbox, for example, can be used as a proxy for true adherence, through capturing box opening by the patient, allowing a granular adherence profile to be generated for each patient.

Understanding factors associated with TB treatment adherence may help identify and/or refine interventions to improve adherence and treatment outcomes. Systematic reviews report variations in the definition of adherence and in the collection of factors associated with adherence [6]. Living with HIV has been found to decrease adherence to TB treatment [6–8]. Among sociodemographic factors, limited access and long distances to health institutions are linked to worse adherence to treatment in Sub-Saharan settings, where HIV prevalence is among the highest globally [9, 10]. Stigmatisation of disease and lack of social support were also stated as reasons for stopping treatment among lost-to-follow-up patients interviewed in Ethiopia [11] and South Africa [12].

The number of large-scale prospective cohort studies measuring adherence to TB treatment and its determinants is limited [4]. In this nested cohort study, we aim to measure the impact of pre-selected baseline determinants on treatment adherence, among adults on a DS-TB regimen, using a large prospective cohort enrolled in cluster-randomised trial of a DAT intervention, conducted in South Africa.

## Methods

This is a secondary analysis of the TB Mate cluster-randomised trial, conducted from 2018 to 2022, which enrolled 2,600 participants being treated for DS-TB [13]. Eighteen clinics from three South African provinces, Gauteng, KwaZulu-Natal, and Western Cape, were randomised to the intervention or control arm. The trial evaluated the effectiveness of a smart pillbox (EvriMED device version 1000) coupled with enhanced care for those with sub-optimal adherence, by measuring treatment adherence and poor treatment outcomes. Participants in the intervention arm used the pillbox to store their medications with a daily audio-visual reminder to take treatment. Real-time data from opening the pillbox (as a proxy for medication intake) were reviewed by study staff and healthcare workers (HCWs), who initiated differentiated care through SMS reminders, calls, and visits for participants. Participants in the control arm were given the pillbox in silent mode and asked to keep their medications in the box with box-opening data only available to the research team (and not HCWs) after the end of the study follow-up. Participants who had a treatment outcome of cured or completed treatment were followed up to 12 months after completing their treatment, with sputum collection to assess for TB recurrence.

Baseline data were collected from a medical record abstraction and participant interviews. Medical history, including method of TB diagnosis, HIV status, and whether participants were on antiretroviral therapy (ART) were abstracted from patients’ clinical records. Data on demographics, risk factors for TB, economic situation, and perceived TB stigma were collected from participants by self-report.

This secondary analysis is limited to the adult participants (≥ 18 years) in the control arm of the trial [13]. Two adherence outcomes were considered, based on patient engagement (or non-engagement) with the pillbox as a proxy for a dose taken: (i) < 80% of pillbox engagement over the treatment period; and (ii) a count of pillbox engagement over the total doses required. If the pillbox malfunctioned on a day, based on a lack of a recorded heartbeat, that day was not considered as part of the total required doses. Similarly, if patients died, the total of required doses was censored at the time of death. Patients lost to follow-up, based on the standard WHO definition, were considered to be non-adherent from the date they were lost to follow-up to the end of the expected 6-month treatment period.

Four exposures were considered a priori: HIV status; ART status among participants living with HIV; time to TB care access; and perceived stigma. HIV and ART status were extracted from clinical records at enrolment and at six months to capture HIV testing and ART initiation at the time of the TB diagnosis. Being on ART was defined as evidence ART was started any time prior and up to 60 days after the start of their TB treatment. The time needed to access clinical care was defined as a combination of self-reported travel time from the participant’s home to the health clinic and the waiting time for the appointment once inside the health clinic, collected at the enrolment visit. Perceived stigma was based on a scale with 10 items self-reported by participants at enrolment. We used the TB stigma scale developed from the ZAMSTAR study in South Africa and Zambia [14]. Due to the low levels of reported stigma, the stigma scale was categorised into a binary variable, to reduce instability of estimates due to sparse data and low prevalence of reported stigma.

To guide the multivariable analyses and the identification of a sufficient set of confounders, Directed Acyclic Graphs (DAGs) were constructed, summarising the perceived direct and indirect pathways between each exposure of interest and the outcome, using Daggity [15, 16]. Age and gender were also considered to be a priori confounders, based on the literature search. Socio-economic position (SEP) was identified as a potential confounder for the four exposures. An asset-based wealth score was constructed with polychoric dual-component analysis, a modified version of principal component analysis, using two principal components to increase the proportion of variance explained by the score and reduce urban bias [17]. Data on housing structure and assets owned were used to develop the wealth score which was grouped into quintiles for analysis.

**Fig. 1:**
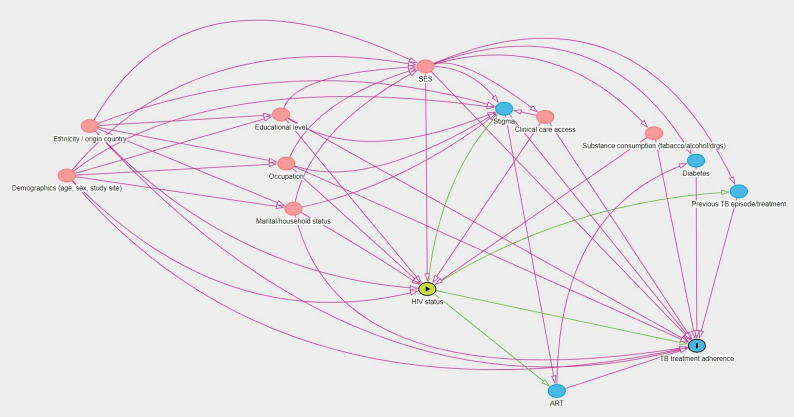
DAG illustrating the causal association between HIV status and the level of adherence to TB treatment. Colour code : the yellow circle indicates the exposure of interest ; red circles indicate the confounders ; blue circles represent the variables considered on the causal pathway, i.e. for which the regression models are not adjusted

The DAG for the causal relationship between HIV status and TB treatment adherence is illustrated in Fig. 1. The following sufficient set of confounders was identified to measure the total causal effect of HIV status: gender, age, clinic, country of origin, ethnicity, education level, occupation, marital status, household status, tobacco, alcohol, and illicit drug consumption, time to access clinical care, and socio-economic position. See Figure S1 for the DAGs for the other a priori exposures. Further details on the development of the DAGs are available in the supplementary materials.

The binary adherence outcome was analysed using logistic regression. The count outcome was analysed using negative binomial regression, as evidence of overdispersion was found, with the total number of days on treatment used as an offset. For both models, fixed-effects to account for the.

clustering at the clinic level was adopted rather than a random effect, given there were only nine clinics. No variables were considered to be a priori effect modifiers, based on a literature approach. For multivariable analyses, four separate regression models were developed for the different exposure variables, as varying sets of confounders were identified based on the DAGs. Due to the minimal proportion of missing values for variables included in the multivariable analyses (< 0.5%), a complete records analysis was conducted. Sensitivity analyses were conducted, with binary poor adherence defined as less than 70% and less than 90% of pillbox engagement. Sensitivity analyses were also conducted with the exclusion of participants lost to follow-up, to observe the potential impact of the assumption that they had stopped taking their treatment.

All analyses were conducted using Stata version 17.0.

The TB Mate trial received ethics approval from the Universities of Witwatersrand (Ref 180705), Cape Town (Ref 452/2018), and the London School of Hygiene and Tropical Medicine (Ref 16107). In addition, ethics approval was obtained from the MSc Research Ethics Committee at the London School of Hygiene and Tropical Medicine (Ref 27519) to conduct this secondary data analysis. The research was conducted in accordance with the Declaration of Helsinki.

## Results

From May 2019 to December 2020, 1,213 adults (range 18–91 years) in the control arm of the TB Mate trial were recruited and followed up. Of these, 742 (61.2%) were male and the median age was 36 years (interquartile range [IQR]: 29–46). The majority, 787 (65.0%), had been diagnosed with TB using GeneXpert MTB/RIF, and 350 (28.9%) had received a clinical diagnosis based on symptoms and clinical signs. 913 (75.3%) were single, not married, at enrolment, and 912 (75.2%) were living with family members, friends, or both. The cost of travelling to the clinic on the day of enrolment was free for 379 individuals (31.2%) and less than 30 South African Rands (ZAR), or 2 US Dollars (1 ZAR = 0.067 USD, 01.01.2022), for an additional 787 (64.9%). 769 participants (63.3%) were living with HIV, of whom 507 were taking ART (65.9%). The median time to access clinical care was 127 min (IQR: 67–188 min). 1,151 participants (96.5%) reported no perceived stigmatisation at the time of starting TB treatment. See Table 1. 76 participants (6.3%) were lost to follow-up during the treatment period.

Overall, 50.6% (614/1213) of participants had adherence of < 80% and the geometric mean of the percentage of pillbox engagement was 59.6% (95%CI: 57.2–62.0).

There was strong evidence that living with HIV was associated with increased odds of poor adherence to TB treatment (odds ratio [OR] 1.49, 95% confidence interval [CI] 1.17–1.90, *p* = 0.001), though taking ART versus not, was not associated with poor adherence (OR 0.90, 95%CI 0.65–1.26, *p* = 0.55). For the other a priori exposures of perceived stigma or time to access clinical care, there was no evidence of an association with poor adherence (Table 1). Being of younger age was associated with worse adherence to TB treatment with strong evidence of a linear trend: each additional year was associated with a 2% reduction in the odds of poor adherence (*p* < 0.001). Results for the count adherence outcome were broadly similar (supplement Table S1).

**Table 1:** Participants’ characteristics and crude odds ratio for poor adherence (< 80%) estimated using logistic regression (*n* = 1213)

	Number of participants (%)*^1^		No. with adherence <80% (%)*^2^		Crude OR (95%CI)		*p*-value*^3^
**HIV status**							
Negative	440	(36.4)	196	(44.6)	1		
Positive	769	(63.6)	415	(54)	1.49	(1.17-1.90)	0.001
**Antiretroviral therapy**							
HIV+ not on ART	262	(34.1)	138	(52.7)	1		
HIV+ on ART	507	(65.9)	277	(54.6)	0.9	(0.65-1.26)	0.55
**Time to access care [min]**							
<60	229	(18.9)	104	(45.4)	1		
60-119	346	(28.5)	172	(49.7)	0.76	(0.45-1.27)	
120-179	299	(24.7)	156	(52.2)	0.76	(0.44-1.30)	
180-239	166	(13.7)	96	(57.8)	1.04	(0.58-1.86)	
>=240	173	(14.3)	86	(49.7)	0.84	(0.46-1.52)	0.45
**Perceived stigma**							
No stigma reported	1,151	(95.2)	580	(50.4)	1		
>=1 point of stigma	58	(4.8)	31	(53.5)	1.05	(0.61-1.82)	0.85
**Age [years]**							
18-25	199	(16.4)	114	(57.3)	1		
26-30	168	(13.9)	96	(57.1)	0.97	(0.64-1.48)	
31-35	211	(17.4)	112	(53.1)	0.84	(0.56-1.24)	
36-40	191	(15.8)	103	(53.9)	0.88	(0.59-1.32)	
41-45	137	(11.3)	62	(45.3)	0.64	(0.41-1.00)	
46-50	123	(10.1)	53	(43.1)	0.56	(0.35-0.89)	
51-60	115	(9.5)	52	(45.2)	0.61	(0.38-0.98)	
>=61	69	(5.7)	22	(31.9)	0.38	(0.21-0.69)	0.008
**Gender**							
Male	742	(61.2)	371	(50)	1		
Female	471	(38.8)	243	(51.6)	1.06	(0.84-1.34)	0.61
**Previous TB episode**							
No	880	(72.6)	426	(48.4)	1		
Yes	333	(27.5)	188	(56.5)	1.36	(1.05-1.77)	0.02
**Mode of TB diagnosis**							
Bact. positive	861	(71.1)	437	(50.8)	1		
Clinical diagnosis	350	(28.9)	162	(46.3)	1.24	(0.95-1.61)	0.11
**Province**							
Gauteng	306	(25.2)	138	(45.1)	1		
Kwa-Zulu Natal	434	(35.8)	216	(49.8)	1.13	(0.68-1.88)	
Western Cape	473	(39)	260	(55)	1.7	(1.01-2.84)	0.09
**Household cohabitants**							
Alone	155	(12.8)	96	(61.9)	1		
Partner/spouse only	146	(12)	64	(43.8)	0.51	(0.32-0.81)	
Family and/or friends	912	(75.2)	454	(49.8)	0.61	(0.42-0.86)	0.008
**Cost for trip to clinic [Rd]**							
Free of charge	379	(31.2)	188	(49.6)	1		
01-Oct	15	(1.2)	19	(66.7)	2.79	(0.90-8.67)	
Nov-20	487	(40.2)	269	(55.2)	1.44	(1.03-2.01)	
21-30	285	(23.5)	130	(45.6)	1.22	(0.79-1.88)	
>=31	47	(3.9)	17	(36.2)	0.75	(0.37-1.51)	0.04

*^1^ Missing values not included: HIV status [4], Antiretroviral therapy [4], Perceived stigma [4], and Previous TB episode [2] // % are column percentages.

*^2^ Number of participants experiencing poor adherence and percentage from the total number of participants with this exposure’s category.

*^3^ P-values calculated using the Likelihood Ratio Test

**Abbreviations**: OR = Odds ratio; CI = Confidence interval; TB = tuberculosis; min = minutes; Rd = South African Rand

In multivariable analyses, there was strong evidence that living with HIV was associated with increased odds of poor adherence (adjusted OR [aOR] 1.68, 95%CI = 1.27–2.22, *p* < 0.001). Similarly to the crude analyses, there was however no evidence that antiretroviral therapy, time to access care, or perceived stigma were associated with poor adherence (Table 2). For adherence modelled as a count of pillbox engagement, there was strong evidence that living with HIV was associated with a 10% lower rate, compared to being HIV-negative (adjusted RR [aRR] 0.90, 95%CI = 0.83–0.97, *p* = 0.005). There was no evidence that receiving ART was associated with better or worse adherence (aRR 0.95, 95%CI = 0.86–1.04, *p* = 0.26). Similarly, there was no evidence that perceived stigma was associated with adherence count (aRR 0.97, 95%CI = 0.84–1.14, *p* = 0.74), or that longer time needed to access care was associated with adherence count.

**Table 2:** Adjusted odds ratios of binary poor adherence and adjusted rate ratios of quantitative adherence to TB

	Outcome : <80% adherence			Outcome : % adherence		*p*-value*^2^
	Adjusted ORs (95%CI)*^1^		*p*-value*^2^	Adjusted RRs (95%CI)*^1^		*p*-value*^2^
Model 1 : HIV status* ^**3**^						
Negative	1			1		
Positive	1.68	(1.27-2.22)	<0.001	0.9	(0.83-0.97)	0.005
Model 2 : ART therapy* ^**4**^						
HIV+ not on ART	1			1		
HIV+ on ART	1.09	(0.75-1.57)	0.65	0.95	(0.86-1.04)	0.26
Model 3 : Time to care [min]* ^**5**^						
<60						
60-119	1			1		
120-179	0.83	(0.48-1.42)		1.02	(0.88-1.18)	
180-239	0.84	(0.47-1.48)		1	(0.86-1.16)	
>=240	1.08	(0.59-1.98)		1	(0.85-1.18)	
Model 4 : Perceived stigma [over 10 pts]* ^**6**^	0.89	(0.48-1.68)	0.73	1.07	(0.90-1.27)	0.87
No stigma reported	1			1		
>=1 point of stigma	1.02	(0.58-1.82)	0.94	0.97	(0.84-1.14)	0.74

*^1^ The number of observations per model slightly differs due to the exclusion of missing variables (as explained in the methods section). The ART models are limited to people living with HIV

*^2^ P-values calculated using the Likelihood Ratio Test

*^3^ Model with 1,209 observations; adjusted for gender, age, clinic, country of origin, ethnicity, education level, occupation, marital status, household status, tobacco, alcohol and illicit drug consumption, time to access clinical care, and socio-economic position

*^4^ Model with 769 observations; adjusted for gender, age, clinic, country of origin, ethnicity, education level, occupation, marital status, household status, previous TB episode, tobacco, alcohol and illicit drug consumption, time to access clinical care, perceived stigma, and socio-economic position

*^5^ Model with 1,213 observations; adjusted for gender, age, clinic, country of origin, ethnicity, education level, occupation, marital status, household status, previous TB episode, HIV status, diabetes, tobacco, alcohol and illicit drug consumption, and socio-economic position

*^6^ Model with 1,209 observations; adjusted for gender, age, clinic, country of origin, ethnicity, education level, occupation, marital status, household status, previous TB episode, tobacco, alcohol and illicit drug consumption, time to access clinical care, HIV status, and socio-economic position

CI confidence interval; OR odds ratio; ART = antiretroviral therapy; RR rate ratio

The sensitivity analysis varying the definition of poor adherence to < 70% (40.8%, 495/1213) and < 90% (64.8%, 786/1213) showed similar results: notably, the strong evidence of worse adherence among participants living with HIV persisted (aOR_70%_ 1.50, 95%CI = 1.13-2.00; aOR_90%_ 1.77, 95%CI = 1.32–2.36) (Table 3). The observed associations remained consistent when excluding the 76 participants (6.3%) who were lost to follow-up on treatment; participants living with HIV had increased odds of poor adherence to TB treatment (OR 1.62, 95%CI 1.21–2.17, *p* = 0.001). The sensitivity analyses for the count outcome gave similar results (data not shown).

**Table 3:** (sensitivity analyses): adjusted odds ratios of poor adherence to TB treatment defined as < 70%, and < 90%^*1^

	<70% adherence		<90% adherence	
	aOR	(95%CI)	aOR	(95%CI)
**HIV status**				
Negative	1		1	
Positive	1.50	(1.13-2.00)	1.77	(1.32-2.36)
**Antiretroviral therapy**				
HIV+ not on ART	1		1	
HIV+ on ART	1.21	(0.84-1.75)	1.13	(0.77-1.67)
**Time to access care [min]**				
60	1		1	
60-119	0.85	(0.50-1.47)	1.12	(0.64-1.96)
120-179	0.88	(0.50-1.56)	1.06	(0.59-1.91)
180-239	1.08	(0.59-1.99)	1.34	(0.71-2.54)
=240	0.95	(0.50-1.79)	1.04	(0.54-1.99)
**Perceived stigma [over 10 pts]**				
No stigma reported	1		1	
=1 point of stigma	1.05	(0.59-1.86)	1.07	(0.58-1.98)

^*1^ Separate logistic regression models for each exposure, adjusted for the same set of confounders and with the same number of observations as regression models in Table 2

CI confidence interval; aOR adjusted odds ratio; ART antiretroviral therapy

## Discussion

In this large South African cohort of 1,213 adults receiving treatment for DS-TB, the geometric mean of the percentage of pillbox engagement was 59.6% (95%CI: 57.2–62.0). We found strong evidence that participants living with HIV had increased odds of poor adherence, and a 10% lower rate of pillbox engagement, compared to those HIV-negative. No evidence of an association was found for ART status, time to clinic, and stigma being associated with adherence. Results also showed that younger age was associated with higher odds of poor adherence.

The absence of association between our exposures and patient engagement with the pillbox, except for HIV status, was not consistent with previous studies [10–12]. This may be due to the differences in design between this cohort study and previous studies conducted on adherence to TB treatment, which were mostly cross-sectional or retrospective by design, and mostly used other means of measuring adherence, such as DOT [18]. Additionally, adherence to TB treatment is linked to complex health behaviours, as well as beliefs linked to the disease, which are strongly context-dependent, and prone to change with time. This may explain that, even within South Africa, determinants of adherence may vary depending on geographical areas and time.

Taking antiretroviral therapy among participants living with HIV was not associated with worse TB treatment adherence, though people living with HIV had lower adherence than those HIV-negative. ART status was determined by ascertaining whether participants living with HIV were prescribed ART within the first two months of TB treatment. However, adherence to ART itself was not recorded and might have been a more sensible exposure variable. As we relied on abstraction from clinic records, ART status may have also been misclassified if ART records were not found.

There was no evidence suggesting that perceived stigma was associated with the outcome either. One issue with this exposure variable was the small number of participants, 58 (4.8%), that reported any stigma at baseline associated with their TB diagnosis. This lowered the statistical power accessible to observe a possible association. In addition, participants were enrolled shortly after their TB diagnosis and stigma was measured at this baseline visit: stigma may have appeared after a certain delay and therefore be missed during this baseline data collection.

Finally, there was no evidence showing that the time needed to access care was linked to treatment adherence, although limited access to healthcare is recognised as a risk factor of poor adherence [9–11]. We hypothesised that participants requiring a longer time to access their clinic would be at increased risk of missing follow-up visits, due to the associated direct and indirect costs, and their disengagement from healthcare would then increase their risk of poor daily adherence. Most participants lived in urban areas relatively close to the clinics (< 8 km away) and needed less than 4 h in total to access care. It may be possible that only a few participants included in this study truly experienced limited access to healthcare, at such a level observed in other studies. However, our measure of access -based solely on travel and waiting time- may not fully reflect other important barriers, such as restrictive clinic operating hours or the perceived quality of care, which could also influence adherence. Moreover, a secondary cost-effectiveness analysis of the TB Mate trial showed that most intervention-related costs were indirect, arising from income lost during clinic visits [19]. Such opportunity costs may have further limited patients’ engagement with the healthcare system.

This study’s sample size, as well as the large number of outcomes recorded, provided good power to measure associations and conduct multivariable regression models. Since there lacks a universal definition of poor adherence to DS-TB treatment, we conducted sensitivity analyses to ensure that our findings remained consistent with different chosen cut-offs for binary adherence. There are, however, some important limitations. Misclassification of the outcome may have occurred: the electronic monitoring device recorded the engagement of participants with the smart pillbox technology and was only a proxy for adherence itself. Voluntary non-adherence from participants, i.e. opening the box without ingesting the medication, would not have been detected. On the other hand, adherence might have also been underestimated, as participants in the control arm had no incentive to use the pillbox to store their medication, and the absence of an independent verification method (e.g., urine isoniazid testing) further limits accuracy. A recent systematic review assessing the performance of digital technologies for measuring TB treatment adherence suggests that some digital technologies, including pillboxes, have suboptimal performance for measuring medication adherence; however, data are limited [20]. A study from India notably showed suboptimal accuracy of DAT engagement (99DOTS) with results from urine isoniazid testing, a direct measure of drug intake [21]. Moreover, because participants were aware that their medication-taking behaviour could be monitored as part of the trial, a Hawthorne effect cannot be excluded, potentially altering dosing intake compared with routine care settings and limiting generalisability. In our study, participants in the control arm had no personal benefit from adherence monitoring by the pillbox and so may have opted not to use the pillbox at all. In addition, participants lost to follow-up were classified as non-adherent from the point of disengagement, which -although assessed in sensitivity analyses- may have overestimated non-adherence in some cases. Our study is restricted to individuals who have initiated TB treatment. Clinic attendees diagnosed with TB but who never started treatment, that is, lost to follow-up before taking their first dose, were excluded. These individuals represent a group with zero adherence, that is extreme non-adherence, and understanding their factors is important. This limits the generalisability of our results, therefore, to a population that actively engages with the local healthcare system, at least once post-treatment initiation.

Although our analysis adjusted for socioeconomic position and baseline stigma, other context-specific factors in South Africa may also influence adherence. Prior studies highlight the role of food insecurity, transport barriers, and migration for work, alongside community-level HIV stigma [22–24]. These structural and cultural dynamics, not fully captured in our quantitative measures, underscore the need for additional qualitative studies.

Sufficient adherence to TB treatment is important to avoid treatment failure or development of new drug resistance, and increased TB burden globally [25]. To best allocate health resources aimed at improving treatment adherence, it is necessary to identify subpopulations at risk of poor adherence. Younger individuals and those living with HIV, comprising a large proportion of this cohort, were identified as having worse DAT engagement, a proxy for adherence to DS-TB treatment. These results highlight the need to bring reinforced differentiated care to this specific population of TB patients in South Africa to improve adherence and ultimately success of TB treatment. Further, qualitative research should also be conducted to understand treatment adherence issues in such groups to determine how best to support their care and treatment. Potentially effective strategies may include enhanced adherence counselling and peer or community-based support, which have been shown to improve both TB and HIV outcomes [26]; integration of TB and HIV services to reduce the burden of multiple clinic visits; and the use of digital adherence technologies, tailored especially to younger individuals and people living with HIV.

## Conclusion

This large cohort study shows that people living with HIV in South Africa have significantly lower adherence to drug-sensitive TB treatment, independently of ART use. Other hypothesised determinants, including time to care and perceived stigma, were not associated with adherence. These findings highlight the need for targeted adherence support for those with TB disease and HIV co-infection to improve treatment outcomes in high-burden settings.

## Supplementary Information

Below is the link to the electronic supplementary material.

Supplementary Material 1

Supplementary Material 2

## Data Availability

The datasets used and/or analysed during the current study are available upon submission of a request to the Aurum Data Governance Committee, on reasonable request.
