# Supplementary material for: Baseline determinants of adherence for drug-sensitive TB treatment in a South African prospective cohort: a focus on HIV infection and anti-retroviral therapy, clinical care access, and TB stigma
Source: BMC Infect Dis. 2026 Jan 7;26:251. doi: 10.1186/s12879-025-12304-4 (PMC12869961; doi:10.1186/s12879-025-12304-4)
Supplement: Supplementary file 1 — Supplementary Material 1 [file 12879_2025_12304_MOESM1_ESM.docx]

***Appendices***

1. Directed acyclic graphs for the other exposure and chosen framework to develop them

Supplement Figure S1: DAG illustrating the causal association between being on ART (among HIV-positive individuals) and the level of adherence to TB treatment


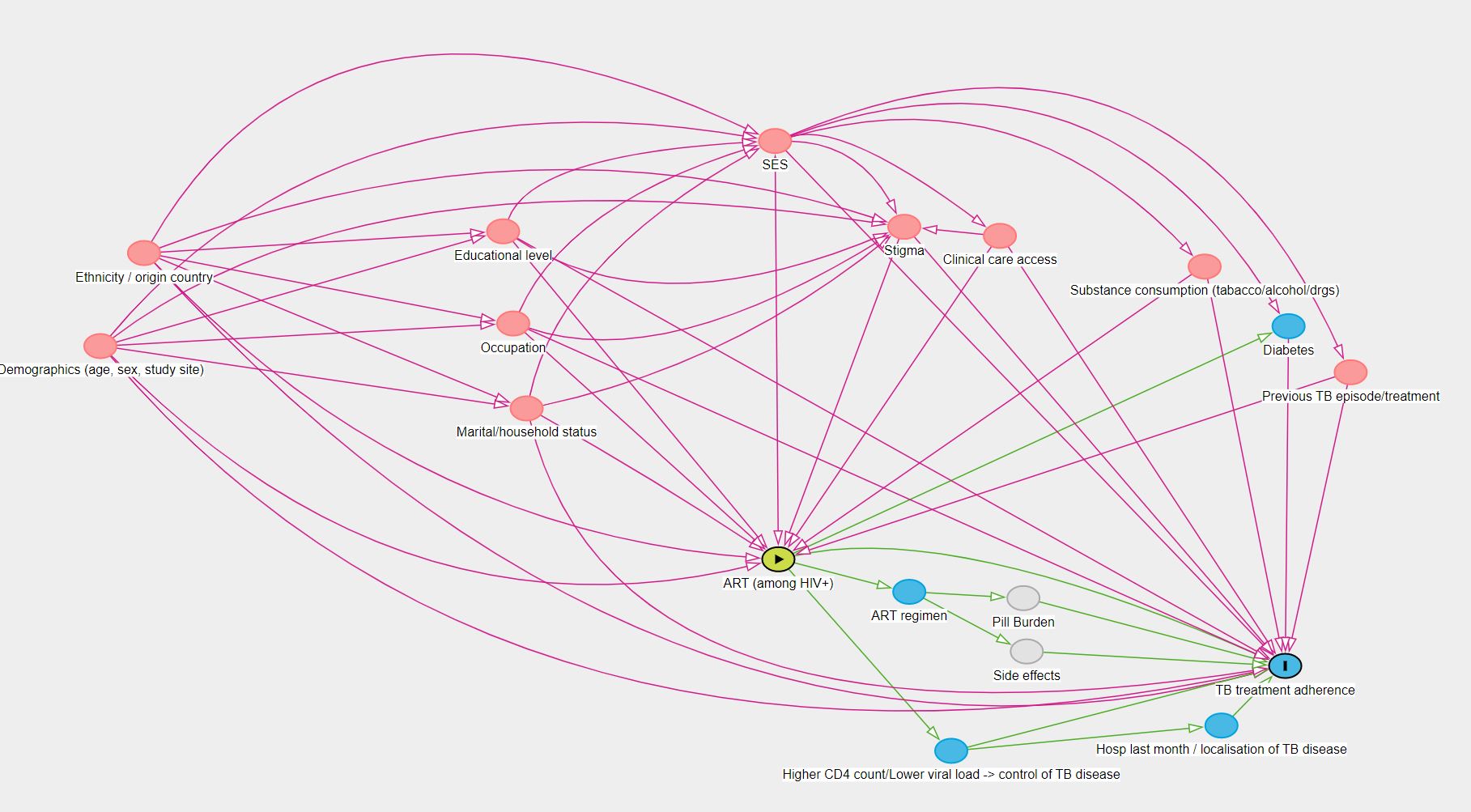


Supplement figure S2: DAG illustrating the causal association between clinical care access and the level of adherence to TB treatment


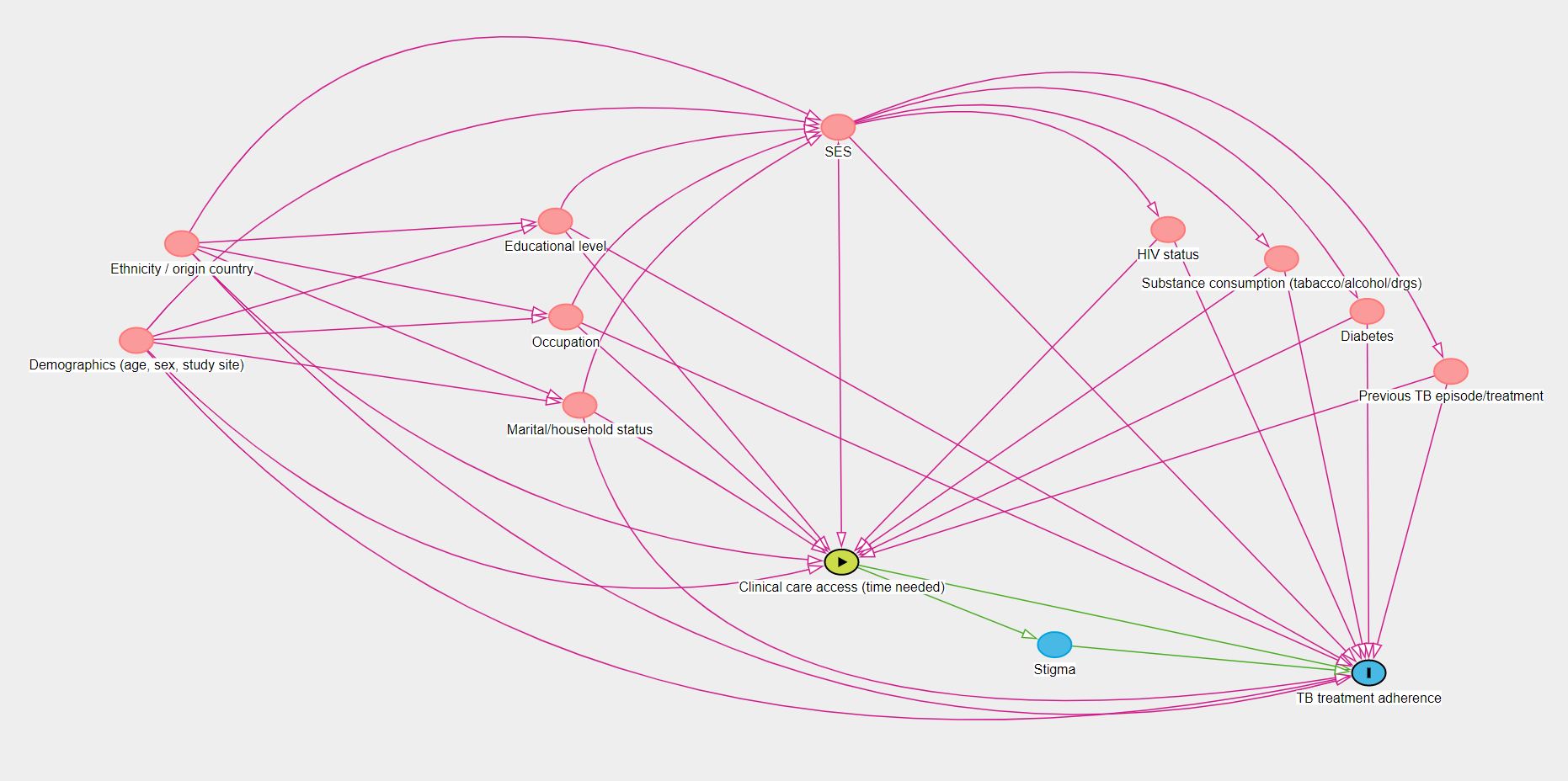


Supplement figure S3: DAG illustrating the causal association self-perceived stigma from TB diagnosis and the level of adherence to TB treatment.


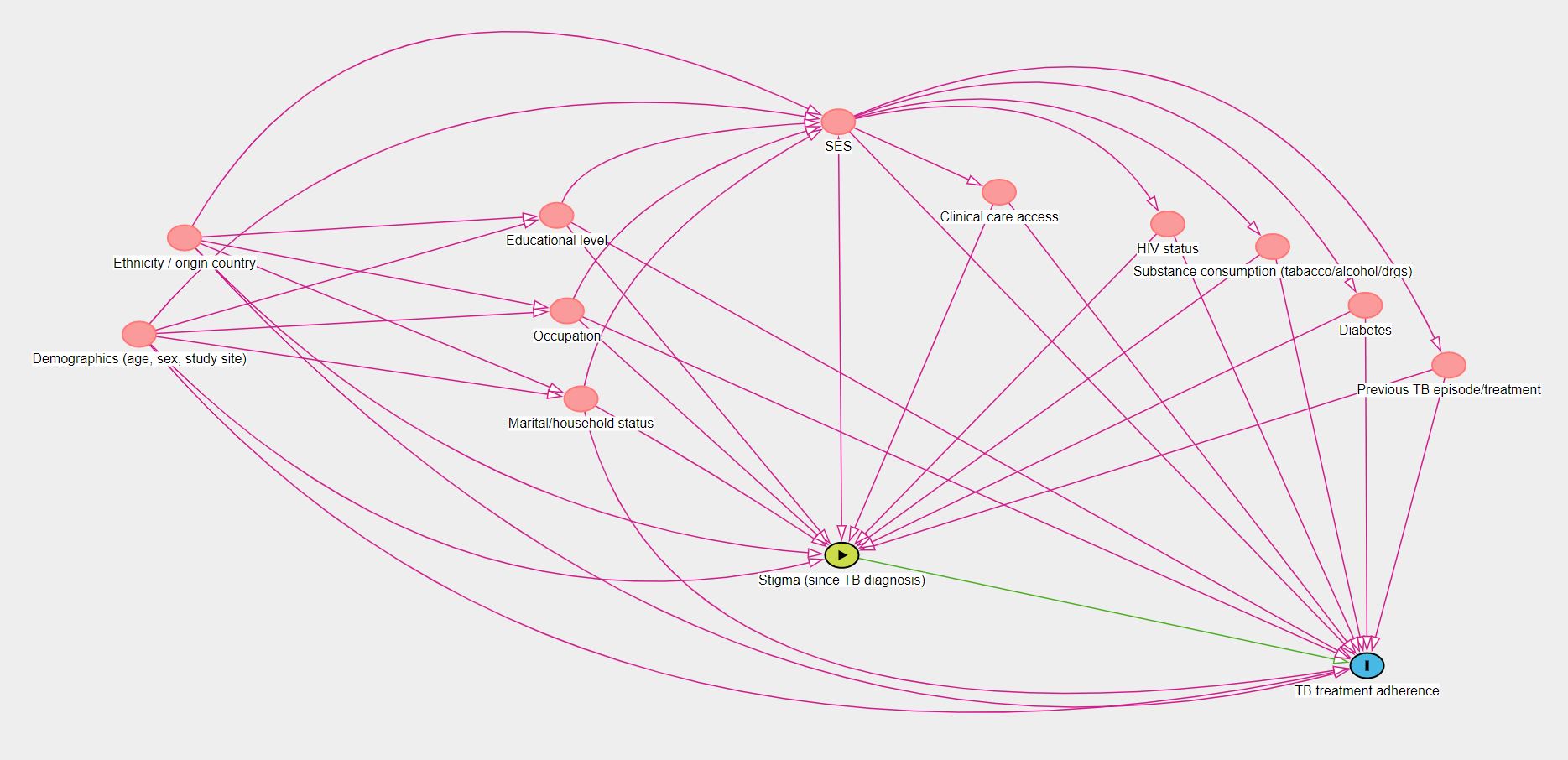


*Framework for the DAGs development:*

To guide the multivariable analyses and the identification of a sufficient set of confounders, Directed Acyclic Graphs were developed, highlighting the direct and indirect pathways between each exposure of interest and the outcome. This also helped to avoid the potential pitfalls linked to the inadequacy of traditional criteria for identifying potential confounders (21).

We used the following reproducible framework to develop each DAG. We first classified the collected covariates as proximal or more distal determinants. We then developed a simple diagram linking the exposure to the outcome and added additional covariates one by one. For each of them, We considered whether they lied on the causal pathway between the exposure and outcome, as well as the direction of their association with the exposure, outcome, and all previously added covariates to the model, using a literature-based approach. The R package “Daggity” was used to build the DAGs, enabling robust causal inference (16). We followed the recommendations for systematicity in the development of DAGs, as proposed in 2021 by Tennant, & al (15).

The DAGs display distal factors on the upper left and more proximal factors towards the lower right. Variables in red are confounders to be conditioned on, variables in blue mediators on the causal pathway, and those in grey non-measured variables. The exposure is displayed in yellow, and the outcome is “TB treatment adherence”. For each of the four DAGs presented below, certain arcs present between confounders were removed, if this did not impact the sufficient set of confounders, in order to improve the visibility of the figures. The following covariates, although accounted for individually in the statistical models, were also regrouped to increase the DAGs’ visibility: ethnicity and country of origin; age, sex, and study site; marital status and household status.

Age and sex were also considered to be a priori confounders, based on the literature search.

1. Equivalent table 1 for the count outcome

Supplement table S1: Geometric mean of the percentage of doses taken and crude rate ratio for count adherence, estimated using negative binominal regression (n=1213)

|  | Geometric mean of % doses taken*^1^ | Crude RR^*2^  (95%CI) |  | p-value*^3^ |
| --- | --- | --- | --- | --- |
| HIV status  Negative  Positive | 63.0  57.7 | 1  0.91 | (0.85-0.98) | 0.013 |
| Antiretroviral therapy  HIV+ not on ART  HIV+ on ART | 59.1  57.0 | 1  0.95 | (0.87-1.05) | 0.34 |
| Time to access care [min]  <60  60-119  120-179  180-239  >=240 | 63.0  60.8  55.7  56.3  63.0 | 1  1.04  1.00  1.00  1.06 | (0.90-1.20)  (0.86-1.17)  (0.85-1.17)  (0.90-1.26) | 0.84 |
| Perceived stigma  No stigma reported  >=1 point of stigma | 59.7  57.6 | 1  0.97 | (0.83-1.12) | 0.65 |
| Age [years]  18-25  26-30  31-35  36-40  41-45  46-50  51-60  >=61 | 55.4  54.6  58.5  60.5  61.2  61.7  66.0  69.1 | 1  1.02  1.05  1.07  1.09  1.12  1.14  1.23 | (0.91-1.14)  (0.94-1.17)  (0.96-1.20)  (0.97-1.24)  (0.99-1.27)  (1.00-1.30)  (1.05-1.43) | 0.15 |
| Gender  Male  Female | 60.3  58.5 | 1  0.99 | (0.93-1.06) | 0.81 |
| Previous TB episode  No  Yes | 60.9  56.2 | 1  0.92 | (0.85-0.99) | 0.02 |
| Mode of TB diagnosis  Bact. positive  Clinical diagnosis | 58.6  62.3 | 1  0.99 | (0.92-1.06) | 0.74 |
| Province  Gauteng  Kwa-Zulu Natal  Western Cape | 59.2  60.9  58.6 | 1  1.01  0.99 | (0.93-1.10)  (0.91-1.08) | 0.85 |
| Household cohabitants  Alone  Partner/spouse only  Family and/or friends | 53.6  64.8  59.9 | 1  1.15  1.11 | (1.02-1.31)  (1.00-1.22) | 0.06 |
| Cost for trip to clinic [Rd]  Free of charge  1-10  11-20  21-30  >=31 | 58.7  66.3  57.8  62.8  64.1 | 1  1.04  0.97  0.99  1.04 | (0.77-1.40)  (0.89-1.07)  (0.88-1.12)  (0.86-1.26) | 0.92 |

*^1^ Missing values not included: HIV status (4), Antiretroviral therapy (4), Perceived stigma (4), and Previous TB episode (2) // % are column percentages

*^2^ Incidence rate ratio of dose intake

*^3^ P-values calculated using the Likelihood Ratio Test

**Abbreviations**: RR=Rate ratio; CI=Confidence interval; TB=tuberculosis; pts=points
