# Supplementary material for: Baseline determinants of adherence for drug-sensitive TB treatment in a South African prospective cohort: a focus on HIV infection and anti-retroviral therapy, clinical care access, and TB stigma
Source: BMC Infect Dis. 2026 Jan 7;26:251. doi: 10.1186/s12879-025-12304-4 (PMC12869961; doi:10.1186/s12879-025-12304-4)
Supplement: Supplementary file 2 — Supplementary Material 2 [file 12879_2025_12304_MOESM2_ESM.docx]

**Abbreviations**

ART : antiretroviral therapy

CI : confidence interval

DAG : directed acyclic graph

DAT : digital adherence technology

DOT : Directly Observed Therapy

DS : drug-sensitive

HCW : healthcare workers

HIV : human immunodeficiency virus

IQR : interquartile range

OR : odds ratio

aOR : adjusted odds ratio

RR : rate ratio

aRR : adjusted rate ratio

SEP : socio-economic position

TB : tuberculosis

USD : US Dollar

WHO : World Health Organization

ZAR : South African Rand
